# The impact of functional training based on clinical nursing pathways informed by evidence-based theory on functional recovery in patients with cerebral hemorrhage

**DOI:** 10.3389/fneur.2025.1558908

**Published:** 2025-05-26

**Authors:** Haijuan Liang, Ping Yuan, Tong Xu, Chao Jin, Cuiling Ji

**Affiliations:** Department of Neurosurgical Intensive Care Unit, Nanjing Drum Tower Hospital, Affiliated Hospital of Medical School, Nanjing University, Nanjing, Jiangsu, China

**Keywords:** cerebral hemorrhage, functional training, clinical nursing pathways, evidence-based, theory

## Abstract

**Background:**

Cerebral hemorrhage significantly impacts patients’ neurological function and daily living capabilities. The study investigates the effect of functional training based on clinical nursing pathways informed by evidence-based theory on the functional recovery in postoperative patients with cerebral hemorrhage.

**Methods:**

This retrospective analysis included 160 patients divided into an intervention group receiving specialized functional training (*n* = 80) and a control group receiving standard care (*n* = 80) from December 2021 to December 2023. Functional recovery was evaluated using the National Institutes of Health Stroke Scale (NIHSS), Modified Barthel Index (MBI), and Glasgow Coma Scale (GCS). Complications and patient satisfaction were also assessed.

**Results:**

The intervention group showed significant improvements in NIHSS, MBI, and GCS scores post-intervention, indicating enhanced neurological function, daily living capabilities, and consciousness levels (*p* < 0.001). Complication rates were lower in the intervention group (2.5%) compared to the control group (18.75%), with statistical significance (*p* = 0.0021). Patient satisfaction was notably higher in the intervention group, with 95% total satisfaction compared to 76% in the control group (*p* < 0.01).

**Conclusion:**

Functional training within clinical nursing pathways, grounded in evidence-based theory, significantly improves postoperative functional recovery, reduces complications, and increases patient satisfaction in individuals recovering from cerebral hemorrhage.

## Introduction

Cerebral hemorrhage, a form of intracranial bleed, constitutes a significant health challenge globally, accounting for a considerable proportion of acute cerebrovascular diseases. It is characterized by the spontaneous rupture of blood vessels within the brain, leading to the accumulation of blood that compresses and damages brain tissues. This condition not only results in immediate neurological deficits but also poses long-term functional impairments (1, 2). The functional recovery in cerebral hemorrhage patients is a multidimensional process that extends beyond mere survival, encompassing the restoration of mobility, cognitive functions, and the ability to perform activities of daily living (3, 4). The efficacy of this recovery process is pivotal in determining the long-term prognosis of patients, making it a primary focus of clinical interventions.

Functional training emerges as a rehabilitation strategy aimed at enhancing the recovery of these functional abilities. It involves tailored exercises and activities designed to mimic daily tasks, thereby facilitating the relearning of motor and cognitive skills affected by the hemorrhage (5, 6). The customization of functional training programs to meet the individual needs of patients is crucial for optimizing recovery outcomes (7). The integration of evidence-based theory into clinical nursing practice has become fundamental to optimize patient outcomes. This approach is characterized by the systematic application of current best evidence from high-quality research, such as randomized controlled trials, clinical guidelines, and meta-analyses, combined with clinical expertise and patient preferences to guide individualized care decisions (8, 9). In the domain of postoperative rehabilitation for cerebral hemorrhage, several studies have demonstrated that evidence-informed interventions, such as goal-oriented motor retraining, early mobilization, and structured cognitive-behavioral engagement, significantly enhance recovery outcomes (10–12). These principles underpin the design of clinical nursing pathways (CNPs), which serve as structured care plans to standardize treatment processes while accommodating patient-specific needs. Therefore, this study aimed to investigate the impact of functional training, structured according to evidence-based Clinical Nursing Pathways, on the postoperative functional recovery of cerebral hemorrhage patients. It contributes to current knowledge by empirically evaluating a theoretically grounded and literature-informed rehabilitation strategy that integrates functional task simulation, progressive mobility training, and early cognitive stimulation.

Although minimally invasive surgical techniques have been widely explored as an approach to improve functional outcomes in cerebral hemorrhage, existing evidence from clinical studies has yielded largely negative or inconclusive results regarding their efficacy in enhancing long-term neurological recovery. This has led to growing interest in non-surgical, rehabilitation-centered approaches. Notably, this study investigated the role of evidence-based clinical nursing pathways, a relatively underexplored yet promising modality, in improving functional recovery post-cerebral hemorrhage. To better delineate its effectiveness, the current study not only compared functional outcomes between standard and evidence-based nursing groups, but also systematically examined differences in intervention content across specific dimensions, such as neurological support, physical rehabilitation intensity, complication prevention strategies, and patient engagement protocols. Moreover, prognosis following cerebral hemorrhage is influenced by multiple clinical variables, including patient age, hemorrhage location, and surgical approach. These factors were considered during the design of this study to ensure homogeneity in baseline characteristics and to minimize confounding effects. Clear and specific inclusion and exclusion criteria were established to enhance the reliability of the findings and to define the patient population eligible for functional training interventions.

## Methods

### Study design

A detailed retrospective analysis was undertaken at our medical facility to assess the influence of function-oriented training, orchestrated within the framework of clinical nursing pathways underscored by evidence-based principles, on the rehabilitation outcomes of individuals diagnosed with cerebral hemorrhage. The review period spanned from December 2021 to December 2023 and included 160 patients who either underwent surgical intervention or received conservative medical treatment, depending on clinical indication. Of these, 80 patients received function-oriented training and were designated as the observation group, while the remaining 80, who received routine care, constituted the control group. Surgical treatment was performed in patients with large hematomas, deteriorating consciousness, brainstem compression, or signs of raised intracranial pressure that did not respond to medical management. The surgical modalities included minimally invasive hematoma evacuation, burr hole drainage, craniotomy for clot evacuation, and in a limited number of cases, decompressive hemicraniectomy or suboccipital craniectomy depending on hematoma location and severity. All participants provided informed consent, and the study protocol received prior approval from the institutional Ethics Committee, ensuring adherence to ethical standards.

### Inclusion and exclusion criteria

Participants were eligible for inclusion if they had a confirmed diagnosis of spontaneous intracerebral hemorrhage (ICH) via CT or MRI imaging, with or without surgical intervention. A crucial criterion is the presence of mobility impairment persisting or becoming evident following initial postoperative stabilization, typically within the first few days post-surgery, making the individual a suitable candidate for structured functional training aimed at rehabilitation. All patients were required to demonstrate cognitive capacity sufficient for providing informed consent and adhering to the rehabilitation regimen, as evaluated by attending neurologists based on cognitive and communicative abilities at the time of enrollment. For patients exhibiting severe neurological impairment (e.g., NIHSS >15), informed consent was obtained from legal guardians or immediate family members, in accordance with institutional Ethics Committee protocols.

To ensure consistency in baseline neurological function and hemodynamic status, participants were included only if they met the following clinical criteria at the time of enrollment:

- NIHSS score between 4 and 15, indicating moderate neurological impairment but preserved capacity for rehabilitation participation.
- GCS score ≥ 13, reflecting intact or minimally impaired consciousness.
- Modified Rankin Scale (mRS) score of 2–4, indicating moderate disability requiring some assistance but with the ability to walk unassisted.
- Stable hemodynamic status, defined as blood pressure and heart rate within normal limits without the need for continuous vasopressor support or intensive monitoring, and the absence of active bleeding or acute deterioration.
- Onset of hemorrhagic event ≥72 h prior to enrollment, to ensure participants were beyond the acute phase of cerebral hemorrhage.
- A single, well-defined hemorrhagic lesion located in the basal ganglia, frontal lobe, or cerebellum, to minimize prognostic variability related to lesion site.
- Surgical intervention, if applicable, performed via craniotomy or minimally invasive hematoma evacuation.
- Documented post-surgical mobility impairment that necessitated functional rehabilitation.
- Age between 45 and 75 years, to exclude pediatric and elderly populations with distinct neuroplasticity characteristics.

#### Exclusion criteria

Participants were excluded if they had:

- Multiple intracerebral hemorrhages or hemorrhage involving the brainstem or ventricles.
- Pre-existing neurological disorders or prior cerebrovascular events (e.g., ischemic stroke, neurodegenerative diseases) that could confound outcome assessment.
- Severe comorbid conditions, such as advanced cardiac disease, end-stage renal or hepatic failure, or terminal malignancies.
- Significant language or communication barriers, including aphasia or language incompatibility, that would impede participation in or evaluation of rehabilitation outcomes.
- Diagnosed severe psychiatric disorders, such as schizophrenia or major depressive disorder, that could impair adherence to the rehabilitation regimen.

To minimize bias, patients who required urgent neurosurgical intervention but succumbed within 48 h of admission were excluded from the analysis, as postoperative rehabilitation could not be feasibly initiated in these cases.

### Nursing protocols for the observation and control groups

Following surgery, the control group received standard postoperative care, which included monitoring vital signs, observing changes in condition, preventing incision infection, and providing medication guidance and health education to both patients and their families. This was complemented by a gradual introduction to rehabilitative exercises. Conversely, the observation group was subjected to Functional Training Based on Clinical Nursing Pathways Informed by Evidence-Based Theory. The implementation was twofold: Initial steps involved identifying factors influencing postoperative recovery from cerebral hemorrhage to pose evidence-based questions. The literature review concentrated on evidence from recent systematic reviews, clinical guidelines (such as those from the American Heart Association and the Chinese Stroke Association), and high-quality interventional studies related to post-stroke rehabilitation and functional recovery (13–16). Key findings informed the development of the nursing pathway and emphasized early mobilization, daily task-specific training, multimodal sensory stimulation, and cognitive rehabilitation. The evidence was appraised and translated into a structured CNP, which delineated phase-specific goals, daily interventions, and expected clinical outcomes. The CNP was reviewed and refined by a multidisciplinary panel consisting of neurologists, rehabilitation specialists, and senior nurses to ensure feasibility and consistency with local clinical practice.

Nursing tasks were executed in accordance with the CNP charts. On the first postoperative day, initial postoperative care was provided, including routine oral hygiene, infection prevention, and optimal bed positioning. In addition, general supportive interventions such as passive limb exercises, joint mobilization, and muscle massage were introduced to prevent complications such as joint stiffness and pressure sores. These early measures were not contingent on confirmed mobility impairment but formed part of standard postoperative care. The assessment of neurological status during this phase helped determine the presence and degree of mobility impairment, guiding the implementation of personalized functional training protocols in subsequent days. By the second day, preliminary rehabilitation outcomes were evaluated to design a targeted, repetitive functional training program. From days 3 to 7, in addition to basic care, patients were guided through early-stage rehabilitation exercises in comfortable positions (either on the unaffected side or supine), involving limb joint flexion, rotation, and full-body muscle massage, with particular attention to pressure areas like bony prominences. Between the 8th day and the second week, patient progress was reviewed to adjust and enhance rehabilitation strategies, focusing on standing balance exercises. This phase transitioned from assisted to independent standing, gradually increasing the duration. Cognitive function training, primarily through repetitive memory exercises, was also introduced. From the third week onward, long-term rehabilitation commenced, incorporating walking exercises, activities of daily living (eating, dressing, grooming), and cognitive and linguistic recovery (reading, storytelling, puzzle-solving). The entire rehabilitative training spanned 4 weeks, divided into three distinct phases to optimize postoperative recovery. The structured intervention was designed to progressively address specific rehabilitation goals—ranging from basic mobility and neurological function to cognitive and functional independence—based on best practices identified through literature review. This phased, individualized approach aimed to optimize recovery by targeting the multifaceted impairments common in post-cerebral hemorrhage patients.

### Postoperative functional recovery assessment metrics

To further investigate the influence of hemorrhage site on neurological recovery, NIHSS scores were stratified by hemorrhage location (i.e., cerebellum, frontal lobe, and basal ganglia) in both pre- and post-intervention phases. This stratification aimed to identify potential differential effects of lesion location on functional improvement trajectories.

Functional recovery was assessed at two time points: upon admission to the hospital (prior to intervention) and at the end of the four-week postoperative intervention period. All assessments were conducted by trained clinical staff using standardized protocols to ensure consistency across evaluations.

- **Neurological Function**: Assessed using the National Institutes of Health Stroke Scale (NIHSS) at admission and 4 weeks post-intervention. The scale was administered by certified neurologists to evaluate the severity of neurological deficits.
- **Daily Living Activities**: The Modified Barthel Index (MBI) was used to measure patients’ independence in daily activities, with evaluations conducted at baseline (post-surgery but before intervention) and again at 4 weeks. Higher scores indicated greater independence.
- **Consciousness Level**: The Glasgow Coma Scale (GCS) was used to assess the level of consciousness at the same two time points. Assessments were performed by nursing staff trained in neurological evaluations.
- **Complications**: Incidence of postoperative complications, including pressure sores, pneumonia, and rebleeding, was monitored and recorded continuously during the four-week follow-up.

#### Nursing satisfaction

Satisfaction was assessed at the conclusion of the intervention period using the Newcastle Satisfaction with Nursing Scale, involving 19 items covering aspects such as nursing service attitude, nurse competency, communication skills, procedural skills, nursing safety management, and health education guidance. Scoring for each item ranges from 1 to 5, with the total possible score varying from 19 to 95 points. Satisfaction levels are categorized as follows: ≥ 77 points indicate very satisfied, 58–76 points indicate satisfied, 39–57 points indicate moderately satisfied, and ≤38 points indicate dissatisfied. Overall satisfaction is calculated using the formula: Satisfaction = [(Number of Very Satisfied + Satisfied cases)/Total number of cases] × 100%. Patients were asked to complete the scale independently or with minimal assistance from nursing staff unfamiliar with their treatment group assignment to minimize bias.

To assess the potential influence of consciousness levels on functional recovery, patients were stratified based on their baseline GCS scores into two categories: impaired consciousness (GCS ≤ 12) and preserved consciousness (GCS > 12). This stratification allowed for the examination of how awareness barriers might modulate the outcomes reflected in NIHSS and MBI scores.

### Statistical analysis

Statistical analyses were executed utilizing the SPSS software, version 27.0, to ensure rigorous data evaluation and interpretation. For the presentation of quantitative variables, the mean and standard deviation (mean ± SD) were employed as central measures of tendency and variability, respectively. The *t*-test, a parametric statistical tool, was applied to compare these variables across different groups, facilitating the identification of any statistically significant differences in means. On the other hand, categorical variables were depicted through frequencies and percentages, offering a clear depiction of the distribution of categorical outcomes within the study population. The Chi-square (χ2) test was utilized to compare these variables across groups, enabling the assessment of the association or discrepancy between categorical variables in different cohorts. The threshold for statistical significance was set at a *p*-value of less than 0.05.

## Results

### Participant analysis

In the observation group, there were 43 males and 37 females, with ages ranging from 46 to 75 years. The mean age was 61.56 ± 3.99 years. The distribution of hemorrhage locations was as follows: 22 cases in the cerebellum, 12 in the frontal lobe, and 46 in the basal ganglia. The duration of hypertension among these patients ranged from 1 to 9 years, with an average duration of 5.16 ± 1.2 years. Regarding educational background, 24 patients had education up to junior high school or below, 36 had completed high school, and 20 had tertiary or higher education. The Body Mass Index (BMI) varied from 18 to 27 kg/m^2^, with an average BMI of 22.68 ± 0.65 kg/m^2^. In the control group, there were 42 males and 38 females, with ages ranging from 45 to 76 years. The average age was 61.15 ± 4.26 years. The hemorrhage locations included 24 cases in the cerebellum, 14 in the frontal lobe, and 42 in the basal ganglia. The hypertension duration ranged from 1 to 10 years, with an average duration of 5.96 ± 1.03 years. Educational levels were distributed as follows: 26 patients had junior high school education or below, 30 had high school education, and 24 had tertiary education or above. The BMI ranged from 17 to 28 kg/m^2^, with an average BMI of 23.15 ± 0.58 kg/m^2^ (Table 1). Comparative analysis of the two groups revealed that general characteristics such as BMI, duration of hypertension, and hemorrhage locations were comparable between the groups (*p* > 0.05), indicating no significant differences in these baseline characteristics.

**Table 1:** Demographic and clinical characteristics of the study population.

Characteristic	Observation group	Control group
Gender (M/F)	43/37	42/38
Age range (years)	46–75	45–76
Mean age (years) ± SD	61.56 ± 3.99	61.15 ± 4.26
Hemorrhage location (cerebellum/frontal lobe/basal ganglia)	22/12/46	24/14/42
Hypertension duration range (years)	1–9	1–10
Mean hypertension duration (years) ± SD	5.16 ± 1.2	5.96 ± 1.03
Education level (junior high or below/high school/tertiary and above)	24/36/20	26/30/24
BMI range (kg/m^2^)	18–27	17–28
Mean BMI (kg/m^2^) ± SD	22.68 ± 0.65	23.15 ± 0.58

### Intervention outcomes on functional recovery

Prior to analyzing overall intervention effects, NIHSS scores were examined by hemorrhage location. In both groups, patients with basal ganglia hemorrhage exhibited the highest baseline NIHSS scores, while those with frontal lobe hemorrhage had moderately elevated scores, and cerebellar cases showed comparatively lower initial scores. Across all hemorrhage sites, post-intervention NIHSS scores demonstrated significant improvement in the observation group relative to the control group (all *p* < 0.01), though the degree of improvement varied by location, with the most pronounced gains observed in patients with cerebellar and frontal lobe hemorrhages. The analysis of pre- and post-intervention functional recovery levels, encompassing both the observation and control groups, yielded significant insights into the efficacy of the interventions applied. The NIHSS scores observed a marked improvement post-intervention in the observation group, indicating a substantial enhancement in neurological function compared to the control group, as evidenced by a *t*-value of 6.49 and a *p*-value less than 0.001. This suggests that the targeted intervention effectively mitigated neurological impairments associated with cerebral incidents. Similarly, the MBI scores, reflecting the patients’ ability to perform daily living activities, showed a significant post-intervention improvement in the observation group. The increase in MBI scores, with a *t*-value of 7.29 and a *p*-value below 0.001, underscores the intervention’s role in fostering greater independence and quality of life for the patients (Table 2). Further analysis was performed to evaluate NIHSS score changes stratified by baseline consciousness levels. In patients with impaired consciousness (GCS ≤ 12), the NIHSS scores showed statistically significant improvements post-intervention, although the magnitude of improvement was smaller than in patients with preserved consciousness (GCS > 12). This suggests that awareness barriers may attenuate the extent of functional recovery achievable through rehabilitation interventions.

**Table 2:** Pre- and post-intervention functional recovery levels in observation and control groups.

Metric	Observation group (*n* = 80)	Control group (*n* = 80)	*t*-value	*p*-value
NIHSS pre-intervention	21.48 ± 3.26	20.78 ± 4.44	1.69	0.093
NIHSS post-intervention	11.47 ± 2.93	15.51 ± 2.27	6.49	<0.001
MBI pre-intervention	40.03 ± 5.50	39.88 ± 7.17	0.16	0.871
MBI post-intervention	59.13 ± 5.87	49.92 ± 6.13	7.29	<0.001
GCS pre-intervention	9.19 ± 1.11	9.32 ± 1.46	0.06	0.951
GCS post-intervention	12.90 ± 0.59	10.83 ± 1.19	9.65	<0.001

NIHSS, National Institutes of Health Stroke Scale; MBI, Modified Barthel Index; GCS, Glasgow Coma Scale.

Furthermore, the GCS scores, used to assess the level of consciousness, demonstrated a notable post-intervention improvement in the observation group. The substantial rise in GCS scores, as indicated by a *t*-value of 9.65 and a *p*-value below 0.001, highlights the intervention’s effectiveness in enhancing cognitive and alertness levels post-cerebral incidents. Contrastingly, the pre-intervention comparisons across NIHSS, MBI, and GCS metrics did not reveal significant differences between the groups, indicating homogeneity in baseline functional levels. This homogeneity underscores the observed post-intervention improvements as a result of the therapeutic interventions rather than pre-existing disparities between the groups (Table 2). In conclusion, the intervention administered to the observation group demonstrated a statistically significant positive impact on postoperative functional recovery, as measured by NIHSS, MBI, and GCS scales. The consistency of improvement across all three functional scales (NIHSS, MBI, and GCS) provides converging evidence that the intervention group experienced a multidimensional enhancement in recovery, not attributable to baseline differences. These statistically significant findings (all *p* < 0.001) support the efficacy of the function-oriented training protocol as an adjunct to routine care.

### Incidence of complications in both patient groups

In the assessment of complication rates post-intervention, the observation group reported 2 cases of pressure sores, while the control group experienced a higher incidence of complications, including 6 cases of pressure sores, 6 instances of pneumonia, and 3 cases of rebleeding. The complication rate in the observation group was notably lower at 2.5%, compared to 18.75% in the control group. This significant disparity in the incidence of complications between the two groups was statistically validated with a χ2 value of 9.48 and a *p*-value of 0.0021. This analysis underscores the effectiveness of the intervention in minimizing postoperative complications, particularly in reducing the occurrence of pressure sores, pneumonia, and rebleeding in patients. While pressure sores, pneumonia, and rebleeding were systematically recorded, other potential complications, such as DVT and urinary tract infections were not comprehensively evaluated due to limitations inherent in the retrospective data collection.

### Analysis of patient satisfaction post-intervention

Following the intervention, a comparative analysis of patient satisfaction levels between the observation and control groups revealed significant findings. The observation group demonstrated a higher proportion of patients reporting very satisfied levels at 30%, compared to 18.75% in the control group. This trend continued with 55% of the observation group’s patients feeling satisfied, in contrast to 40% within the control group. Although a smaller percentage of patients in the observation group were moderately satisfied (10%) compared to the control group (17.5%), the dissatisfaction rate was markedly lower in the observation group at 5%, against 23.75% in the control group. When considering the total satisfaction, which encompasses very satisfied and satisfied responses, the observation group exhibited a higher rate at 95%, compared to 76% in the control group. The statistical analysis underpinning these observations, indicated by a Chi-square (χ2) value of 9.95 and a *p*-value less than 0.01, underscores the significant difference in satisfaction levels between the two groups post-intervention (Table 3). These findings highlight the effectiveness of the intervention strategies implemented in the observation group, leading to enhanced patient satisfaction post-treatment.

**Table 3:** Patient satisfaction levels post-intervention.

Satisfaction level	Observation group (*n* = 80)	Control group (*n* = 80)	χ2 value	*p*-value
Very satisfied	24 (30%)	15 (18.75%)	–	–
Satisfied	44 (55%)	32 (40.0%)	–	–
Moderately satisfied	8 (10%)	14 (17.5%)	–	–
Dissatisfied	4 (5%)	19 (23.75%)	–	–
Total satisfaction	76 (95%)	61 (90%)	9.95	<0.01

## Discussion

In the realm of cerebral hemorrhage management, the postoperative phase is crucial for determining long-term outcomes and quality of life. The intricate interplay between physical recovery and the prevention of secondary complications presents a significant challenge in the clinical setting. Functional training, when meticulously designed and implemented within clinical nursing pathways, emerges as a pivotal strategy to address these challenges (10). This approach, informed by evidence-based theory, ensures that interventions are not only scientifically grounded but also tailored to meet the individual needs of patients. The integration of functional training into clinical nursing pathways represents a confluence of rehabilitation principles and nursing care, emphasizing a holistic approach to patient recovery (11, 12). Such training, which encompasses exercises and activities aimed at restoring mobility, strength, and daily living skills, is paramount for patients recovering from cerebral hemorrhage. The adoption of evidence-based practices within this framework ensures that the interventions are aligned with the latest research findings, optimizing recovery outcomes (13, 14). The study’s results elucidate the multifaceted benefits of the implemented interventions in the observation group, manifesting in enhanced functional recovery, reduced complication rates, and elevated patient satisfaction.

The significant post-intervention improvements in NIHSS, MBI, and GCS scores within the observation group can be attributed to a targeted and individualized approach to rehabilitation. The enhanced neurological function, as reflected by NIHSS scores, likely stems from the neuroplasticity of the brain. Neuroplasticity, the brain’s ability to reorganize itself by forming new neural connections, can be significantly influenced by targeted rehabilitation exercises that encourage the recruitment of adjacent, uninjured brain areas to compensate for damaged regions (15, 16). This principle underpins many modern rehabilitation strategies and may explain the observed improvements in neurological function. The improvement in MBI scores, indicative of better performance in daily living activities, suggests that the interventions effectively addressed motor and cognitive deficits (17). However, it is important to note that patients with impaired consciousness exhibited comparatively attenuated improvements in NIHSS scores. This observation aligns with prior research indicating that diminished awareness can interfere with the active participation required for neurorehabilitation, thereby limiting the benefits of functional training. The GCS-based stratification underscores the need for tailored interventions in this subgroup, potentially incorporating more intensive sensory stimulation and prolonged recovery timelines. Rehabilitation techniques such as task-specific training, motor imagery, and cognitive-behavioral therapy could have played crucial roles. These methods are known to enhance motor recovery and cognitive function by leveraging the principles of repetitive practice and goal-oriented tasks, which are essential for re-establishing the neural circuits responsible for these functions. The rise in GCS scores post-intervention points towards improved consciousness levels, possibly facilitated by the integration of cognitive and sensory stimulation into the rehabilitation process (18). Sensory enrichment through auditory, visual, or tactile stimuli can activate cortical areas and promote cognitive engagement, contributing to heightened alertness and orientation.

The lower incidence of pressure sores, pneumonia, and rebleeding in the observation group could be attributed to several interrelated factors. Firstly, the comprehensive approach to patient care, encompassing regular monitoring, early mobilization, and patient education, might have played a pivotal role. Regular repositioning and the use of pressure-relieving devices can significantly reduce the risk of pressure sores by alleviating prolonged pressure on vulnerable areas. Early mobilization, a key component of the interventions, not only aids in functional recovery but also minimizes the risk of pneumonia by enhancing pulmonary function and preventing stasis of pulmonary secretions (19). Moreover, the adherence to evidence-based guidelines for postoperative care, including optimal control of blood pressure and meticulous surgical techniques, could have contributed to the reduced incidence of rebleeding.

The higher satisfaction levels reported in the observation group may be attributed to the holistic and patient-centered approach adopted by the healthcare team. Effective communication, empathy, and the involvement of patients and their families in the care process are critical components of patient-centered care, known to significantly impact satisfaction. The personalized nature of the interventions, tailored to meet the individual needs and preferences of each patient, likely fostered a sense of involvement and empowerment among patients, contributing to their overall satisfaction (3, 20). Furthermore, the reduced complication rates and improved functional outcomes undoubtedly played a role in enhancing patient satisfaction. Patients’ perceptions of care quality are often influenced by their recovery trajectory, with fewer complications and better functional outcomes translating into higher satisfaction levels.

## Limitations

Despite the promising findings, this study has several limitations that warrant consideration. First, the retrospective design may introduce selection bias, limiting the ability to establish causality between interventions and outcomes. Second, the study’s sample size, while adequate, may not fully capture the diversity of the patient population affected by cerebral hemorrhage, potentially affecting the generalizability of the results. Additionally, while NIHSS, MBI, and GCS provide robust assessments of neurological, functional, and consciousness status, they may not fully capture nuanced variations in patient engagement due to fluctuating levels of awareness. The impact of impaired consciousness—particularly in patients with low GCS—on rehabilitation responsiveness highlights the need for more refined tools and layered analysis in future research. While causality could not be definitively established due to the retrospective design, the methodological rigor, including baseline equivalence and multiple validated outcome measures, could enhance the credibility of the observed associations. Future studies should address these limitations through prospective designs, larger and more diverse samples, and a broader range of outcome measures to provide a more holistic understanding of the impact of functional training on postoperative recovery in cerebral hemorrhage patients. The inclusion of hemorrhage size, location, and laterality in future studies will provide further insights into their potential impact on the outcomes of rehabilitation programs, particularly in terms of cognitive, linguistic, and motor recovery. Laterality of the hemorrhage, especially in regions, such as the frontal lobe, plays a crucial role in determining the patient’s capacity to engage in functional training and rehabilitation, impacting overall recovery.

## Conclusion

In conclusion, incorporating functional training within clinical nursing pathways informed by evidence-based theory significantly enhances neurological function and daily living capabilities in patients’ post-cerebral hemorrhage. This approach not only mitigates complications but also accelerates recovery, ultimately leading to heightened patient satisfaction. Such evidence-based interventions are crucial for optimizing postoperative outcomes and improving the quality of life for these patients.

## Data Availability

The original contributions presented in the study are included in the article/supplementary material, further inquiries can be directed to the corresponding author.

## References

[1] Hostettler IC Seiffge DJ Werring DJ. Intracerebral hemorrhage: an update on diagnosis and treatment. Expert Rev Neurother. (2019) 19:679–94. doi: 10.1080/14737175.2019.1623671 31188036

[2] Ziai WC Carhuapoma JR. Intracerebral hemorrhage. Continuum (Minneap Minn). (2018) 24:1603–22. doi: 10.1212/CON.0000000000000672, PMID: 30516598

[3] Fu X Hu C Fu J Li Z. Integrated nursing and targeted functional training for cerebral hemorrhage recovery. Altern Ther Health Med. (2023) 29:80–5. PMID: 37499153

[4] Li Q Chen L. Effect of tibialis anterior muscle resistance training on ankle and foot dorsum extension function in hypertensive cerebral hemorrhage hemiplegia patients: a randomized controlled trial. Medicine (Baltimore). (2023) 102:e33827. doi: 10.1097/MD.0000000000033827, PMID: 37543805 PMC10403024

[5] Øie LR Madsbu MA Solheim O Jakola AS Giannadakis C Vorhaug A. Functional outcome and survival following spontaneous intracerebral hemorrhage: a retrospective population-based study. Brain Behav. (2018) 8:e01113. doi: 10.1002/brb3.1113, PMID: 30240164 PMC6192392

[6] Schepers VP Ketelaar M Visser-Meily AJ de Groot V Twisk JW Lindeman E. Functional recovery differs between ischaemic and haemorrhagic stroke patients. J Rehabil Med. (2008) 40:487–9. doi: 10.2340/16501977-0198, PMID: 18509566

[7] Knecht S Hesse S Oster P. Rehabilitation after stroke. Dtsch Arztebl Int. (2011) 108:600–6. doi: 10.3238/arztebl.2011.0600, PMID: 21966318 PMC3183303

[8] Okuda Y Aoike F. Functional recovery of patients with intracerebral haemorrhage and cerebral infarction after rehabilitation. Int J Rehabil Res. (2021) 44:222–5. doi: 10.1097/MRR.0000000000000476, PMID: 34034286

[9] Saulle MF Schambra HM. Recovery and rehabilitation after intracerebral hemorrhage. Semin Neurol. (2016) 36:306–12. doi: 10.1055/s-0036-1581995, PMID: 27214706 PMC5324055

[10] Uniken Venema SM Marini S Lena UK Morotti A Jessel M Moomaw CJ. Impact of cerebral small vessel disease on functional recovery after intracerebral hemorrhage. Stroke. (2019) 50:2722–8. doi: 10.1161/STROKEAHA.119.025061, PMID: 31446887 PMC6756971

[11] Chen Y Zhang Q You N Wang L. Analysis of influencing factors of neurological function recovery and cerebral hemorrhage transformation after intravenous thrombolysis in patients with acute ischemic stroke. Zhonghua Wei Zhong Bing Ji Jiu Yi Xue. (2020) 32:1340–5. doi: 10.3760/cma.j.cn121430-20200713-00517, PMID: 33463494

[12] Chu CL Chen YP Chen CCP Chen CK Chang HN Chang CH. Functional recovery patterns of hemorrhagic and ischemic stroke patients under post-acute care rehabilitation program. Neuropsychiatr Dis Treat. (2020) 16:1975–85. Published 2020 Aug 13. doi: 10.2147/NDT.S253700, PMID: 32884273 PMC7431596

[13] Sun L Jing S Zhang L. Combined effect of early physical exercise and stereotactic hematoma evacuation in patients with cerebral hemorrhage undergoing hemodialysis. Altern Ther Health Med. (2023) 29:347–51. PMID: 37652407

[14] Zhao S Zhang T Zhao J Li B Wu Z. A retrospective analysis of factors impacting rehabilitation outcomes in patients with spontaneous intracerebral hemorrhage. Am J Phys Med Rehabil. (2020) 99:1004–11. doi: 10.1097/PHM.0000000000001459, PMID: 32371627

[15] Baig AA Monteiro A Waqas M Cappuzzo JM Siddiqi M Doane J. Acute isolated posterior cerebral artery stroke treated with mechanical thrombectomy: a single-center experience and review of the literature. Interv Neuroradiol. (2023) 29:10–9. doi: 10.1177/15910199211070949, PMID: 35001703 PMC9893240

[16] Hays SA Khodaparast N Hulsey DR Ruiz A Sloan AM Rennaker RL 2nd. Vagus nerve stimulation during rehabilitative training improves functional recovery after intracerebral hemorrhage. Stroke. (2014) 45:3097–100. doi: 10.1161/STROKEAHA.114.006654, PMID: 25147331 PMC4175144

[17] Massicotte S Lun R Yogendrakumar V Dewar B Chung HS Konder R. How outcomes are measured after spontaneous intracerebral hemorrhage: a systematic scoping review. PLoS One. (2021) 16:e0253964. doi: 10.1371/journal.pone.0253964, PMID: 34191862 PMC8244847

[18] Long F Qin K Liao S Wu J Tang C Liu T. Individualized treatment of intraventricular hemorrhage guided by modified Graeb criteria score and Glasgow coma scale. Zhonghua Wei Zhong Bing Ji Jiu Yi Xue. (2019) 31:1373–7. doi: 10.3760/cma.j.issn.2095-4352.2019.11.012, PMID: 31898568

[19] Stoltenberg S Kotila J Heikkilä A Kvist T Junttila K. Incidence and risk factors for pressure injuries in adults in specialised medical care: a prospective observational study. J Wound Care. (2021) 30:945–53. doi: 10.12968/jowc.2021.30.11.945, PMID: 34747213

[20] Jin Z Guo F Li Y. Effects of systemic rehabilitation nursing combined with WeChat publicity and education on the early cognitive function and living quality of the patients with cerebral arterial thrombosis. J Healthc Eng. (2022) 2022:7396950–7. doi: 10.1155/2022/7396950, PMID: 35251575 PMC8894030

